# Supplementary material for: Host-microbe co-metabolism via MCAD generates circulating metabolites including hippuric acid
Source: Nat Commun. 2023 Jan 31;14:512. doi: 10.1038/s41467-023-36138-3 (PMC9889317; doi:10.1038/s41467-023-36138-3)
Supplement: Supplementary file 1 — Supplementary Information [file 41467_2023_36138_MOESM1_ESM.pdf]

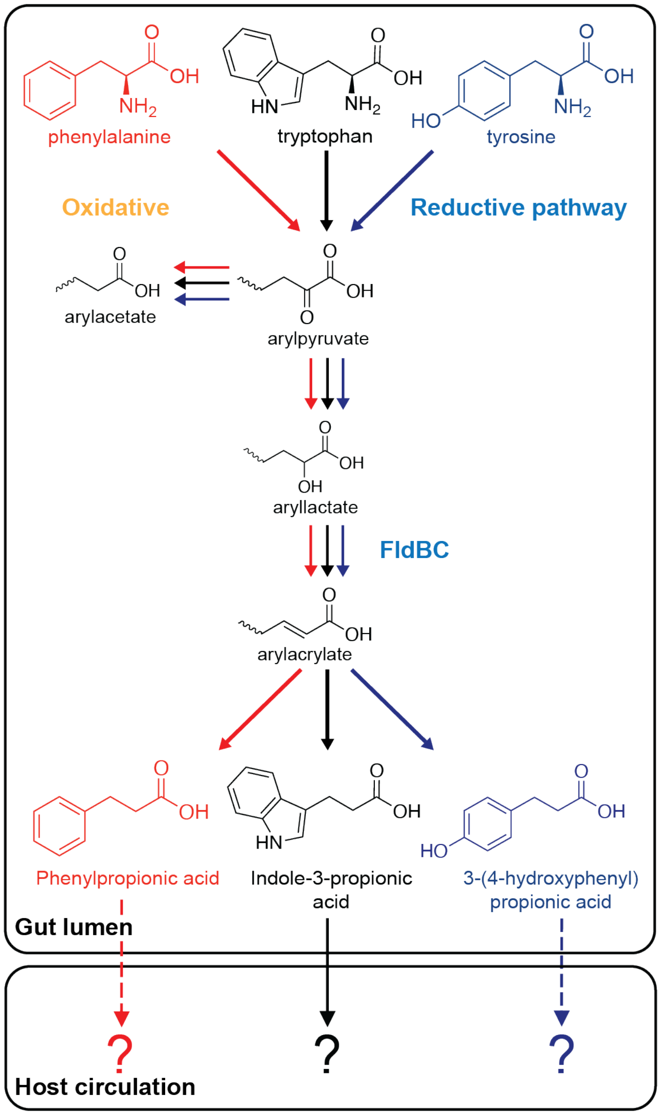

Supplementary Fig. 1: Pathways for aromatic amino acid metabolism in *C. sporogenes*. Indolepropionic acid, the product of tryptophan metabolism is found at high levels in host circulation and influences gut barrier integrity and immune function. Potential host effects and levels in circulation of 3-phenylpropionic acid and 3-(4-hydroxyphenyl)propionic acid, the products of phenylalanine and tyrosine metabolism respectively, are unknown.

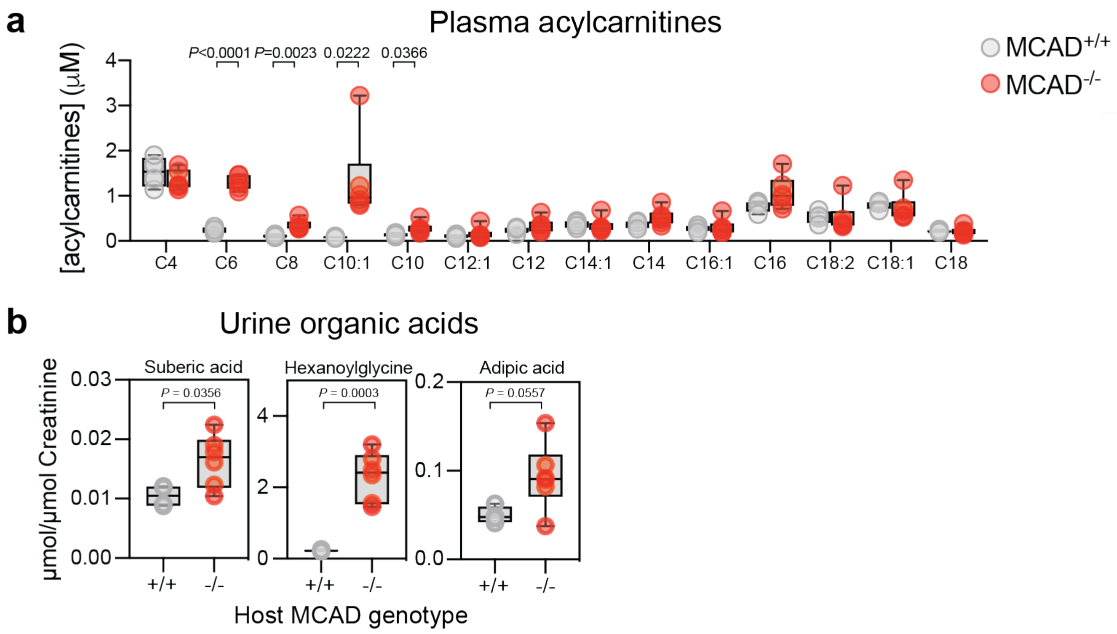

Supplementary Fig. 2: Phenotypic verification of MCAD$^{-/-}$ mouse model. GF MCAD$^{-/-}$ and MCAD$^{+/+}$ mice were fasted for 24 hours prior to plasma (**a**) and urine (**b**) collection. **a,** MCAD$^{-/-}$ mice exhibit significantly higher C6-C10 acylcarnitines in serum, as determined by MS/MS of their butyl-ester derivatives (two-tailed unpaired t-tests with Welch's correction). **b,** Creatinine-normalized LC-MS-based quantification of urine organic acids (suberic acid, $C_8H_{14}O_4$; adipic acid, $C_6H_{10}O_4$) and hexanoylglycine, which were previously shown at higher abundance in MCAD$^{-/-}$ mice (two-tailed unpaired Student's t-tests). For **a** and **b,** n=4 MCAD$^{+/+}$ and 6 MCAD$^{-/-}$ mice; boxes denote the median with inter-quartile distance, whiskers denote maxima and minima.

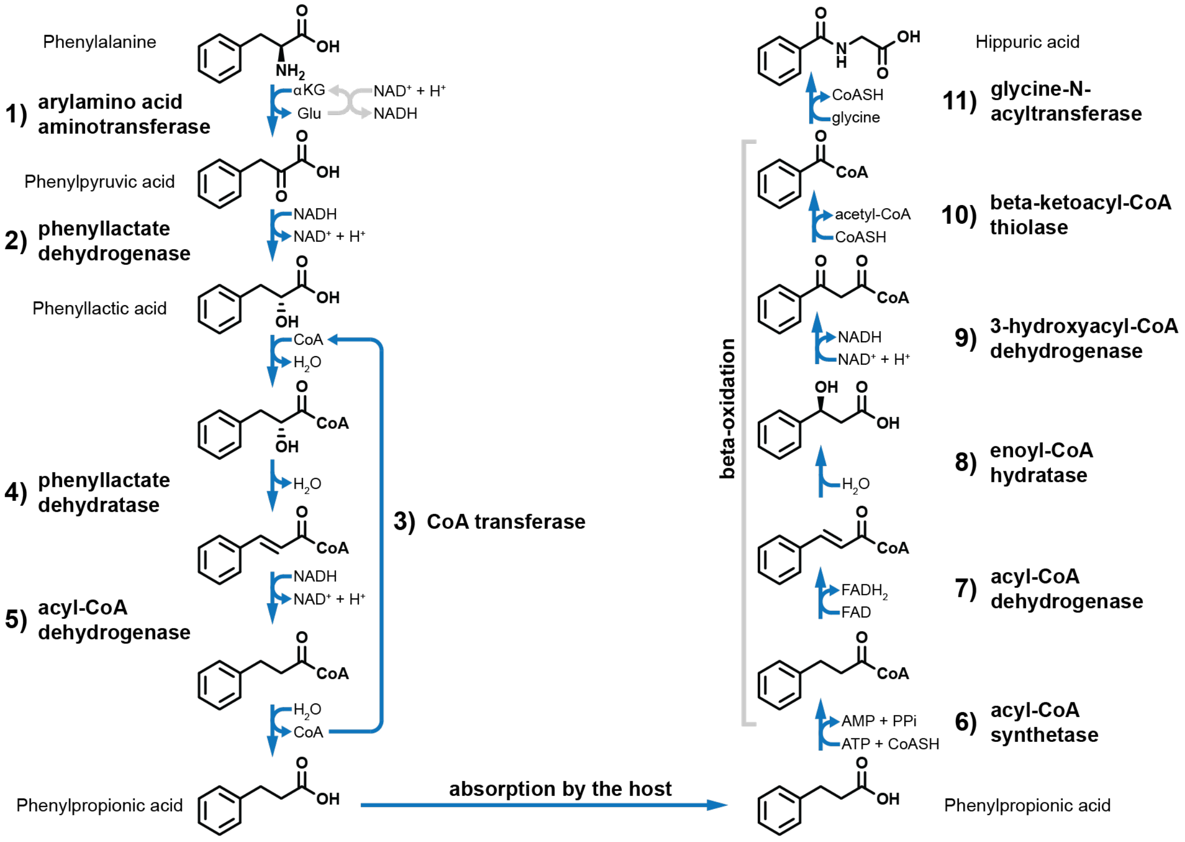

Supplementary Fig. 3: Proposed eleven-step pathway for host-microbe conversion of phenylalanine to hippuric acid. Steps 1 through 5 are catalyzed by bacteria in the gut, involving reductive metabolism of phenylalanine to phenylpropionic acid. Phenylpropionic acid is absorbed and undergoes one round of β-oxidation followed by glycine conjugation to produce hippuric acid.

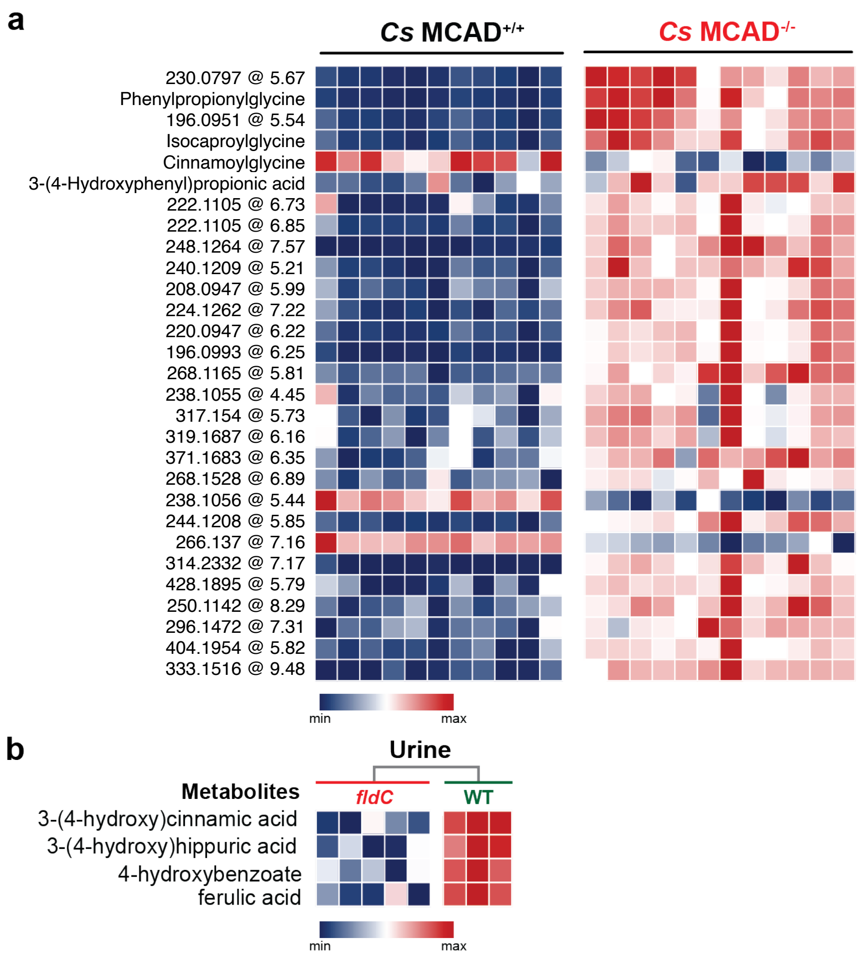

Supplementary Fig. 4: Urinary metabolites differentiate mice based on a functional MCAD gene. a, Metabolites significantly different between MCAD$^{-/-}$ and MCAD$^{+/+}$ mouse urine that depend on *C. sporogenes* colonization. For unnamed metabolites, m/z with retention time (minutes) is given. Each column represents creatinine-normalized peak area for an individual mouse, scaled to row. **b,** Metabolites that differed significantly based on *C. sporogenes fldC* and were uniquely different in urine (multiple unpaired two-tailed t-tests with two-stage linear step-up procedure of Benjamini, Krieger, and Yekutieli, adjusted *P*-value < 0.05). Each column represents row-normalized peak intensity for an individual mouse.
