# Supplementary material for: Host-microbe co-metabolism via MCAD generates circulating metabolites including hippuric acid
Source: Nat Commun. 2023 Jan 31;14:512. doi: 10.1038/s41467-023-36138-3 (PMC9889317; doi:10.1038/s41467-023-36138-3)
Supplement: Supplementary file 3 — Description of Additional Supplementary Files [file 41467_2023_36138_MOESM3_ESM.docx]

**File Name: Supplementary Data 1.**

Description: GC-TOF metabolomics of WT vs. *fldC* mono-colonized mice.

**File Name: Supplementary Data 2.**

Description: Significant discoveries from GC-TOF metabolomics of WT vs. *fldC*-colonized mice. Multiple two-tailed unpaired t-tests were conducted: individual variance was calculated for each metabolite in each host compartment, multiple comparisons were corrected for with the false discovery rate (FDR) calculated via the Benjamini, Krieger, and Yekutieli two-stage step-up method.

**File Name: Supplementary Data 3.**

Description: LC-MS metabolomics of MCAD^+/+^ vs. MCAD^-/-^ mice.

**File Name: Supplementary Data 4.**

Description: MS/MS spectra for samples and their standards shown in Figure 5b.
