# Supplementary material for: Host-microbe co-metabolism via MCAD generates circulating metabolites including hippuric acid
Source: Nat Commun. 2023 Jan 31;14:512. doi: 10.1038/s41467-023-36138-3 (PMC9889317; doi:10.1038/s41467-023-36138-3)
Supplement: Supplementary file 8 — Reporting Summary [file 41467_2023_36138_MOESM8_ESM.pdf]

nature portfolio

Corresponding author(s): Dylan Dodd
Last updated by author(s): 1/6/2022

Reporting Summary

Nature Portfolio wishes to improve the reproducibility of the work that we publish. This form provides structure for consistency and transparency in reporting. For further information on Nature Portfolio policies, see our Editorial Policies and the Editorial Policy Checklist.

Statistics

For all statistical analyses, confirm that the following items are present in the figure legend, table legend, main text, or Methods section.

n/a	Confirmed	
☐	☒	The exact sample size (*n*) for each experimental group/condition, given as a discrete number and unit of measurement
☐	☒	A statement on whether measurements were taken from distinct samples or whether the same sample was measured repeatedly
☐	☒	The statistical test(s) used AND whether they are one- or two-sided<br>*Only common tests should be described solely by name; describe more complex techniques in the Methods section.*
☒	☐	A description of all covariates tested
☐	☒	A description of any assumptions or corrections, such as tests of normality and adjustment for multiple comparisons
☐	☒	A full description of the statistical parameters including central tendency (e.g. means) or other basic estimates (e.g. regression coefficient) AND variation (e.g. standard deviation) or associated estimates of uncertainty (e.g. confidence intervals)
☐	☒	For null hypothesis testing, the test statistic (e.g. *F*, *t*, *r*) with confidence intervals, effect sizes, degrees of freedom and *P* value noted<br>*Give P values as exact values whenever suitable.*
☒	☐	For Bayesian analysis, information on the choice of priors and Markov chain Monte Carlo settings
☒	☐	For hierarchical and complex designs, identification of the appropriate level for tests and full reporting of outcomes
☐	☒	Estimates of effect sizes (e.g. Cohen's *d*, Pearson's *r*), indicating how they were calculated

Our web collection on statistics for biologists contains articles on many of the points above.

Software and code

Policy information about availability of computer code

Data collection	Mass spectrometry data were collected using MassHunter LC/MS Data Acquisition version 10.1 (Agilent technologies, Inc.).
Data analysis	Mass spectrometry data were analyzed using Agilent MassHunter Quantitative Analysis (version 10.0) Agilent MassHunter Qualitative Analysis (version 10.0), MS-DIAL (version 4.24) Microsoft Excel, and GraphPad Prism versions 8 and 9.

For manuscripts utilizing custom algorithms or software that are central to the research but not yet described in published literature, software must be made available to editors and reviewers. We strongly encourage code deposition in a community repository (e.g. GitHub). See the Nature Portfolio guidelines for submitting code & software for further information.

Data

Policy information about availability of data

All manuscripts must include a data availability statement. This statement should provide the following information, where applicable:

- Accession codes, unique identifiers, or web links for publicly available datasets
- A description of any restrictions on data availability
- For clinical datasets or third party data, please ensure that the statement adheres to our policy

The untargeted mass spectrometry data generated in this study have been deposited in the Metabolomics Workbench database. Raw GC-TOF shotgun metabolomics data is available under the accession number ST001606; untargeted LC-MS data, ST001633. Our analyses of these datasets are provided in Supplementary Tables 1-4; all remaining data generated in this study are provided in the Source Data file.

Human research participants

Policy information about studies involving human research participants and Sex and Gender in Research.

Reporting on sex and gender	N/A
Population characteristics	N/A
Recruitment	N/A
Ethics oversight	N/A

Note that full information on the approval of the study protocol must also be provided in the manuscript.

Field-specific reporting

Please select the one below that is the best fit for your research. If you are not sure, read the appropriate sections before making your selection.

[x] Life sciences [ ] Behavioural & social sciences [ ] Ecological, evolutionary & environmental sciences

For a reference copy of the document with all sections, see nature.com/documents/nr-reporting-summary-flat.pdf

Life sciences study design

All studies must disclose on these points even when the disclosure is negative.

Sample size	No statistical methods were used to predetermine sample sizes. Group sizes were determined based on availability of sex- and age-matched animals due to gnotobiotic breeding and housing constraints.
Data exclusions	In Fig 2c, one outlier at t=3 hours (55.88 µM d5-hippuric acid) was tested for and removed with the Extreme Studentized Deviate method implemented in GraphPad Prism 8. The outlier removed was conservative and its removal altered the distribution of the data at 3 hours in a direction that did not further support the hypothesis. Raw data including the outlier is provided in the Source Data file.
Replication	Gnotobiotic mouse experiments shown were performed once, except for the data shown in Figures 5d and 5f, which were repeated twice.
Randomization	The order of GC-TOF and LC/MS-MS samples were randomized prior to running on the respective instruments.
Blinding	As no subjective measures were employed in this study, researchers were not blinded to groups during sample collection from mice nor during metabolite quantitation during mass spectrometry.

Reporting for specific materials, systems and methods

We require information from authors about some types of materials, experimental systems and methods used in many studies. Here, indicate whether each material, system or method listed is relevant to your study. If you are not sure if a list item applies to your research, read the appropriate section before selecting a response.

Materials & experimental systems

n/a	Involved in the study	
x		Antibodies
x		Eukaryotic cell lines
x		Palaeontology and archaeology
	x	Animals and other organisms
x		Clinical data
x		Dual use research of concern

Methods

n/a	Involved in the study	
x		ChIP-seq
x		Flow cytometry
x		MRI-based neuroimaging

Animals and other research organisms

Policy information about studies involving animals; ARRIVE guidelines recommended for reporting animal research, and Sex and Gender in Research

Laboratory animals	Mouse experiments were performed on gnotobiotic Swiss Webster germ-free mice (males, 10-14 weeks of age, n = 3-8 per group for

	all experiments) or Swiss-Webster Excluded Flora mice ("conventional mice", males, 10-14 weeks of age, n = 3 per group), maintained in aseptic isolators, and originally obtained from Taconic Bioscience. Mice were maintained on a 12-hour light/dark cycle at 69 F in ambient humidity, fed ad libitum, and maintained in flexible film gnotobiotic isolators for the duration of all experiments (Class Biologically Clean, Madison WI).
Wild animals	No wild animals were used in this study.
Reporting on sex	Each experiment comparing colonization state and/or host genotype was performed with sex-matched animals. Both male and female mice were used; the sex of animals used in a given experiment was determined by animal availability within our gnotobiotic facility.
Field-collected samples	No field-collected samples were used in this study.
Ethics oversight	All animal experiments were performed in accordance with the Stanford Institutional Animal Care and Use Committee.

Note that full information on the approval of the study protocol must also be provided in the manuscript.
